# Correction: Dynamic response study of Ti_3_C_2_-MXene films to shockwave and impact forces

**DOI:** 10.1039/d1ra90005f

**Published:** 2021-02-04

**Authors:** Shreyas Srivatsa, Pavithra Belthangadi, Shivakarthik Ekambaram, Manu Pai, Prosenjit Sen, Tadeusz Uhl, Saurabh Kumar, Krzysztof Grabowski, M. M. Nayak

**Affiliations:** Academic Center for Materials and Nanotechnology (ACMiN), AGH University of Science and Technology (UST) Krakow Poland sshreyas@agh.edu.pl; Centre for Nano Science and Engineering (CeNSE), Indian Institute of Science (IISc) Bangalore India saurabh2203@iisc.ac.in; Atomic, Molecular and Optical Physics Division, Physical Research Laboratory Ahmedabad India; Instrumentation and Applied Physics, Indian Institute of Science (IISc) Bangalore India; Department of Robotics and Mechatronics, AGH University of Science and Technology Krakow Poland

## Abstract

Correction for ‘Dynamic response study of Ti_3_C_2_-MXene films to shockwave and impact forces’ by Shreyas Srivatsa *et al.*, *RSC Adv.*, 2020, **10**, 29147–29155, DOI: 10.1039/D0RA04879H.

The authors regret that there was an error in the affiliations of authors Tadeusz Uhl and Krzysztof Grabowski in the original article. The correct list of affiliations is given in this document.

In addition, there was an error in the axis labels for Fig. 8a. The units of the *y*-axis should read mN rather than N. The correct version of the figure is given here. The results and conclusions presented in the article are unaffected by this change.
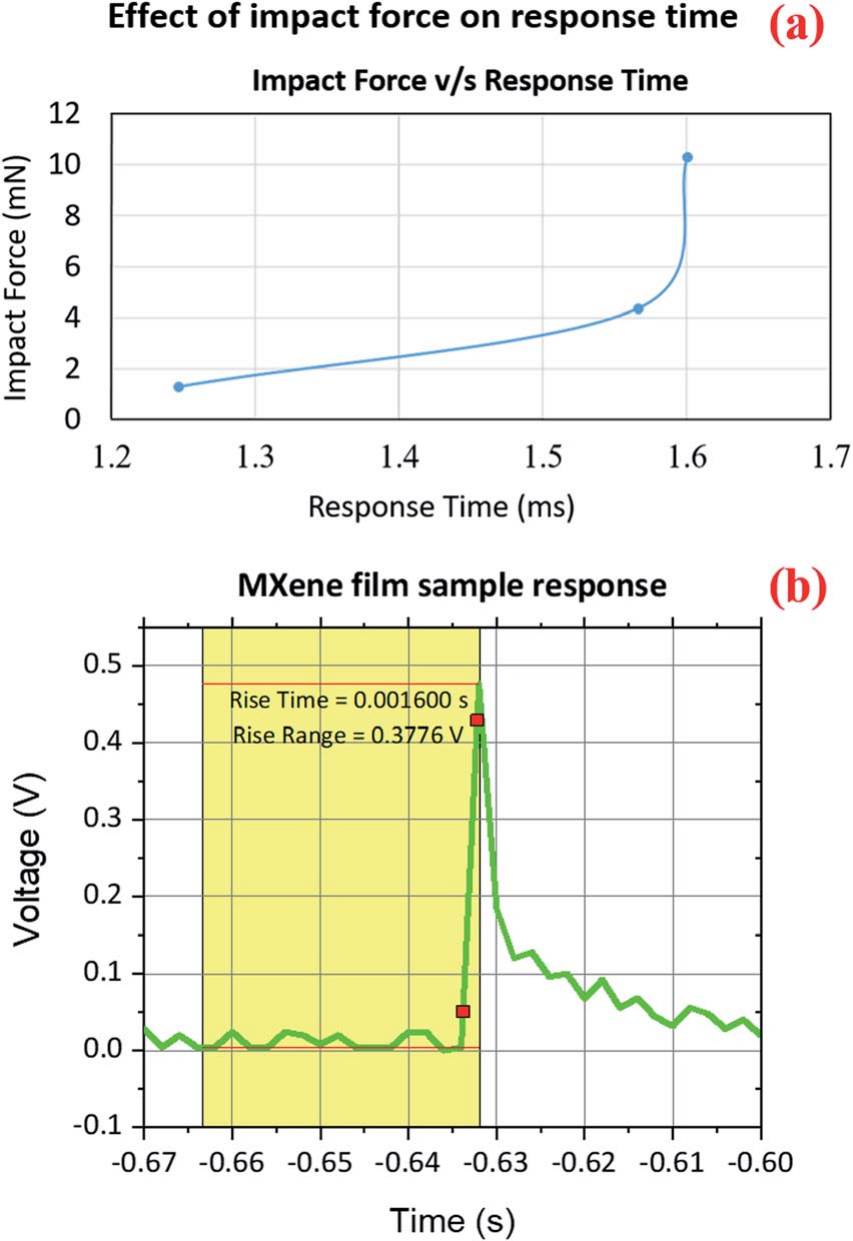


The Royal Society of Chemistry apologises for these errors and any consequent inconvenience to authors and readers.

## Supplementary Material

